# Dynamical Models of the Interactions Between Vaccination and Antibiotic Resistance

**DOI:** 10.3390/vaccines14030212

**Published:** 2026-02-26

**Authors:** Ruoyu Zhang, Jianping Zhu

**Affiliations:** 1 National Institute for Data Science in Health and Medicine, Xiamen University, Xiamen 361005, China; 2 School of Medicine, Xiamen University, Xiamen 361005, China; 3 School of Management, Xiamen University, Xiamen 361005, China

**Keywords:** *Streptococcus pneumoniae*, antibiotic resistance, SEVIR dynamical model, mathematical modelling

## Abstract

**Background/Objectives**: *Streptococcus pneumoniae* remains a major cause of invasive disease, and antimicrobial resistance is shaped by antibiotic selection and pneumococcal conjugate vaccination. A unified framework is needed to compare proposed mechanisms that maintain coexistence of antibiotic-sensitive and -resistant strains and to interpret post-vaccine resistance trajectories. **Methods**: We formulated a susceptible–exposed–vaccinated–infectious–recovered (SEVIR) transmission model that tracks antibiotic-sensitive and -resistant pneumococcal infections under vaccination and treatment. The basic reproduction number ($R_{0}$) was derived using the next-generation matrix method and used to assess local stability of the disease-free equilibrium. Using the same core structure, we evaluated three mechanism-specific extensions: treatment diversity (heterogeneous antibiotic use across host groups), pathogen diversity (serotype/subtype heterogeneity under vaccine targeting), and treatment competition (within-host competition with treatment-induced selection). **Results**: Treatment diversity generated stable coexistence by creating low-treatment refugia that counterbalanced strong selection in highly treated groups, supporting resistance persistence at moderate population-average treatment. Pathogen diversity reproduced serotype-specific replacement and concentration of resistance within particular subtypes after vaccination. Treatment competition produced nonlinear responses to antibiotic intensity and transient resistance surges. Overall, each mechanism explained a distinct subset of benchmark resistance patterns, suggesting that dominant drivers depend on epidemiological context. **Conclusions**: Interactions between vaccination, antibiotic pressure, population heterogeneity, pathogen diversity and within-host competition can yield qualitatively different resistance dynamics. Strategies combining high vaccine uptake with targeted antibiotic stewardship are likely required to curb resistance while limiting unintended serotype replacement.

## 1. Introduction

*Streptococcus pneumoniae* remains a leading cause of pneumonia, meningitis, and invasive disease worldwide, particularly among children and the elderly [1,2]. The widespread use of pneumococcal conjugate vaccines (PCVs) has markedly reduced the burden of vaccine-type (VT) disease; however, non-vaccine serotypes (NVTs) have expanded in many regions, a phenomenon commonly referred to as serotype replacement [3,4,5]. Concurrently, increasing reliance on antibiotics has contributed to the global rise in drug-resistant pneumococcal strains [6,7]. These opposing selective pressures—vaccination and antimicrobial use—interact in complex ways, shaping competition between drug-sensitive and drug-resistant strains across heterogeneous host populations.

Mathematical models have been widely used to explore pneumococcal transmission and the evolution of antimicrobial resistance. Existing studies have provided important insights into vaccine-induced serotype dynamics, antibiotic-driven selection for resistance, and competition between pneumococcal strains [8,9,10,11]. However, most current models focus on isolated mechanisms and often assume homogeneous host populations or uniform pathogen characteristics. Only a limited number of studies explicitly consider how vaccination reshapes the selective environment for resistance [12,13], and even fewer incorporate differences in antibiotic use or treatment practices across host subpopulations [14]. As a result, the combined effects of (i) host-level treatment heterogeneity, (ii) pathogen diversity reflected by sensitive and resistant strains, and (iii) antibiotic-induced competition remain poorly understood within a unified theoretical framework.

Antibiotic selection pressure plays a dual role in pneumococcal dynamics. While treatment reduces the duration and transmissibility of drug-sensitive infections, it simultaneously promotes the competitive release of resistant strains [15,16]. Such treatment competition can generate nonlinear feedback loops, particularly when resistant strains carry fitness costs or when vaccination disproportionately reduces transmission of drug-sensitive serotypes [12,17]. In real populations, antibiotic exposure is highly heterogeneous, varying across age groups, clinical settings, and socioeconomic strata. This treatment diversity creates distinct selective environments that collectively influence the persistence and spread of resistance [14,18]. Capturing these interacting mechanisms requires models that integrate multiple host subgroups, multiple strain types, and both vaccination and treatment dynamics.

In this study, we develop a unified SEVIR-based dynamical framework for pneumococcal transmission that simultaneously incorporates treatment diversity, pathogen diversity, and treatment competition. We begin with a baseline SIR formulation and progressively extend it to include vaccination, antibiotic treatment, sensitive and resistant strains, and heterogeneous host groups. Using the next-generation matrix approach, we derive explicit expressions for the basic reproduction number and invasion conditions for drug-resistant strains [19]. We characterize the disease-free equilibrium, analyze its stability, and identify parameter regimes that permit coexistence or dominance of resistance. Sensitivity analyses and numerical simulations further illustrate how vaccination and treatment interact to shape resistance dynamics. By integrating multiple biological and behavioral mechanisms into a mathematically rigorous framework, this study provides theoretical insights into how vaccination and antibiotic use jointly influence pneumococcal resistance evolution and may help inform strategies for managing antimicrobial resistance in the context of ongoing immunization programs.

## 2. Materials and Methods

### 2.1. Baseline SEVIR Model

Extending the classical SIR formulation, we introduce an exposed class (E) to capture a short establishment stage between exposure and detectable/established carriage. We consider a population structured into six epidemiological states: susceptible (S), vaccinated (V), exposed (E), infected with antibiotic-sensitive strain ($I_{S}$), infected with antibiotic-resistant strain ($I_{R}$), and recovered (R). Antibiotic treatment affects only sensitive infections.

The baseline dynamics are given by the following ODE system:

(1) $$\left\{\begin{array}{l} \frac{dS}{dt}=-\beta_{S}SI_{S}-\beta_{R}SI_{R}-\nu S\\ \frac{dV}{dt}=\nu S-q(\beta_{S}VI_{S}+\beta_{R}VI_{R})\\ \frac{dE}{dt}=\beta_{S}SI_{S}+\beta_{R}SI_{R}+q(\beta_{S}VI_{S}+\beta_{R}VI_{R})-\sigma E\\ \frac{dI_{S}}{dt}=(1-\theta )\sigma E-\gamma_{S}I_{S}-\tau I_{S}\\ \frac{dI_{R}}{dt}=\theta \sigma E-\gamma_{R}I_{R}\\ \frac{dR}{dt}=\gamma_{S}I_{S}+\gamma_{R}I_{R} \end{array}\right.$$

where $\beta_{R}$ represents the transmission rate of the drug-resistant strain, $\beta_{S}$ represents the transmission rate of the drug-sensitive strain, q represents the vaccine failure rate, σ represents the progression rate from the exposed to the infectious class, θ the probability that an infected individual becomes resistant during treatment, τ effective selection pressure; $\gamma_{S}$ the natural recovery rate for drug-sensitive infections, and $\gamma_{R}$ the natural recovery rate for drug-resistant infections.

τ represents an effective antibiotic exposure (or treatment pressure) at the population level, which aggregates (i) the probability that a colonized individual develops symptomatic disease, (ii) the probability of receiving antibiotics when symptomatic, and (iii) the average duration/intensity of therapy.

This system forms the foundation upon which all subsequent model extensions are constructed.

### 2.2. Model Extension

To evaluate how different biological and behavioral forces shape resistance dynamics, we extend the baseline SEVIR model along three dimensions: treatment diversity (TD): variation in antibiotic use across subpopulations, pathogen diversity (PD): intrinsic differences across pathogen serotypes, and treatment competition (TC): within-host competition and treatment-induced conversion. Each extension adds a distinct structural heterogeneity without altering the model’s core framework.

#### 2.2.1. Treatment-Diversity Extension

**Figure 1:**
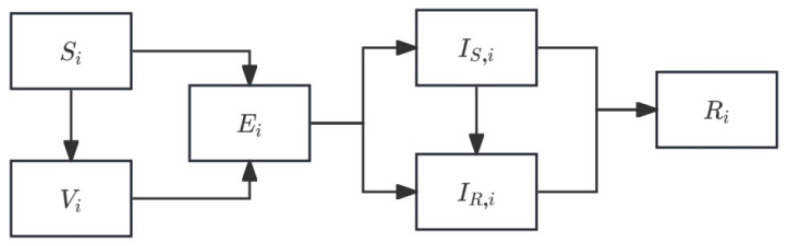
Schematic representation of the treatment diversity model. Individuals in subpopulation i progress through susceptible, vaccinated, early carriage establishment, drug-sensitive infection, drug-resistant infection, and recovery compartments. All epidemiological processes are identical across subpopulations except for the antibiotic treatment rate, which removes drug-sensitive infections at different intensities. The schematic highlights how heterogeneous treatment pressure alone can generate differential resistance dynamics across subpopulations.

Treatment diversity assumes that the host population is divided into subgroups that differ in their antibiotic treatment intensity. Such heterogeneity generates distinct selective environments for sensitive and resistant strains and is well supported by recent theoretical and empirical studies [14]. In high-treatment subpopulations, sensitive infections are rapidly cleared, increasing selective pressure favoring resistant lineages, whereas low-treatment groups act as long-term reservoirs that sustain sensitive–resistant coexistence. This mechanism has been repeatedly demonstrated in evolutionary epidemiology models, showing that heterogeneous antibiotic exposure alone is sufficient to maintain resistance at the population level despite reductions in overall antibiotic consumption [14,18]. Furthermore, spillover between groups with different treatment rates amplifies resistance even in populations with modest aggregate antibiotic use [18,20]. Therefore, the treatment-diversity extension captures a key empirical pattern: persistent coexistence of sensitive and resistant strains in populations with unequal access to antibiotics. A schematic representation of the treatment-diversity model is shown in Figure 1.

Compared with the baseline SEVIR model, the only modification introduced in the treatment-diversity extension is the subgroup-specific antibiotic treatment rate, while all transmission and progression parameters remain identical across subpopulations. The host population is partitioned into K subgroups indexed by i, each characterized by treatment rate and vaccination coverage. Infection and treatment processes occur separately in each subgroup, but mixing in the force of infection is shared at the population level.

For subgroup i, the extended system becomes:

(2) $$\left\{\begin{array}{l} \frac{dS_{i}}{dt}=-\beta_{S}S_{i}I_{S,i}-\beta_{R}S_{i}I_{R,i}-\nu_{i}S_{i}\\ \frac{dV_{i}}{dt}=\nu_{i}S_{i}-q(\beta_{S}V_{i}I_{S,i}+\beta_{R}V_{i}I_{R,i})\\ \frac{dE_{i}}{dt}=\beta_{S}S_{i}I_{S,i}+\beta_{R}S_{i}I_{R,i}+q(\beta_{S}V_{i}I_{S,i}+\beta_{R}V_{i}I_{R,i})-\sigma E_{i}\\ \frac{dI_{S,i}}{dt}=(1-\theta_{i})\sigma E_{i}-\gamma_{s}I_{S,i}-\tau_{i}I_{S,i}\\ \frac{dI_{R,i}}{dt}=\theta_{i}\sigma E_{i}-\gamma_{R}I_{R,i}\\ \frac{dR_{i}}{dt}=\gamma_{S}I_{S,i}+\tau_{i}I_{S,i}+\gamma_{R}I_{R,i} \end{array}\right.$$

This extension captures the epidemiological consequences of heterogeneous antibiotic exposure while maintaining shared transmission pools.

#### 2.2.2. Pathogen-Diversity Extension

Pathogen diversity captures the ecological and evolutionary differences among circulating pathogen subtypes, such as pneumococcal serotypes, which are known to vary substantially in transmissibility $\beta_{j}$, duration of carriage, resistance propensity $\theta_{j}$, and vaccine susceptibility. To represent pathogen diversity, we extend the baseline SEVIR framework to D circulating serotypes/subtypes. Throughout the analysis, D is a free parameter; we use D = 3 as a default illustrative setting to visualize subtype-level dynamics and to facilitate comparison across scenarios. A large body of genomic and epidemiological evidence demonstrates that serotype-specific fitness differences shape both pre- and post-vaccine epidemiology [21,22,23]. Highly fit serotypes dominate transmission, whereas lower-fitness serotypes persist via niche partitioning or frequency-dependent selection [22,23]. Vaccination further reshapes pathogen structure by disproportionately reducing vaccine-targeted serotypes, thereby promoting expansion of non-vaccine serotypes with higher baseline transmissibility or greater resistance burden [4,21,23].

**Figure 2:**
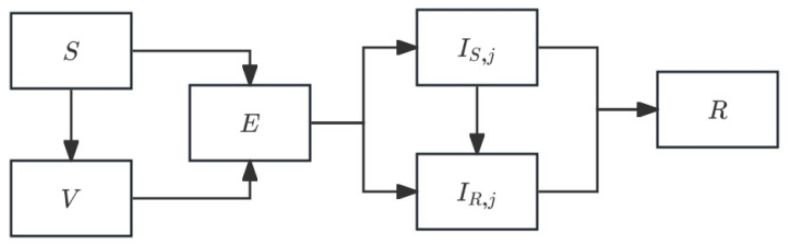
The diagram illustrates the structure of the pathogen diversity model, in which susceptible (S), vaccinated (V), and early carriage establishment (E) individuals are shared across all pathogen subtypes. Each subtype j possesses its own drug-sensitive and drug-resistant infection compartments, governed by subtype-specific transmission rates, resistance development probabilities, and recovery rates. All infected states ultimately transition to the recovered class (R). The model captures how strain-specific ecological advantages can drive coexistence and serotype replacement.

Compared with the baseline SEVIR model, the pathogen-diversity extension introduces subtype-specific infection compartments and parameters $\beta_{j}$, $\theta_{j}$, $\gamma_{S,j}$, $\gamma_{R,j}$, while retaining a shared susceptible, vaccinated, and exposed pool. Hosts thus experience a common force of infection, but once infected, individuals follow subtype-specific epidemiological trajectories that reflect intrinsic fitness differences. This framework explains serotype replacement, uneven resistance distribution across serotypes, and the long-term coexistence of sensitive and resistant variants within the same pathogen lineage. A schematic representation of the treatment-diversity model is shown in Figure 2.

The extended system for subtype j is:

(3) $$\left\{\begin{array}{l} \frac{dS}{dt}=-\sum_{j=1}^{D}[\omega_{j}\beta_{j}S(I_{S,j}+(1-c_{j})I_{R,j})]-\nu S\\ \frac{dV}{dt}=-q\sum_{j=1}^{D}[\beta_{j}V(I_{S,j}+I_{R,j})]+\nu S\\ \frac{dE}{dt}=\sum_{j=1}^{D}[\beta_{j}(S+qV)(I_{S,j}+I_{R,j})]-\sigma E\\ \frac{dI_{S,j}}{dt}=(1-\theta_{j})\omega_{j}\sigma E-\gamma_{S,j}I_{S,j}-\tau I_{S,j}\\ \frac{dI_{R,j}}{dt}=\theta_{j}\omega_{j}\sigma E-\gamma_{R,j}I_{R,j}\\ \frac{dR}{dt}=\sum_{j=1}^{D}\left[\gamma_{S,j}I_{S,j}+\tau I_{S,j}+\gamma_{R,j}I_{R,j})\right] \end{array}\right.$$

This formulation allows serotype-specific differences in transmissibility, resistance tendency, and fitness cost.

#### 2.2.3. Treatment-Competition Extension

The treatment-competition extension captures ecological feedbacks that arise when drug-sensitive and drug-resistant strains interact within hosts and compete for transmission opportunities. Unlike the treatment-diversity and pathogen-diversity extensions, which introduce heterogeneity at the host or pathogen level, this mechanism focuses on within-host competitive processes and treatment-induced selection, both of which have been strongly supported by recent theoretical and empirical work [15,16,17,23].

Antibiotic treatment suppresses sensitive infections, reducing their transmission and creating ecological space—known as competitive release—that allows resistant strains to expand [17]. At the same time, coexistence of sensitive and resistant strains within the same host enables frequency-dependent interactions that stabilize long-term coexistence [15,23]. These interactions are captured in the model by two co-colonization compartments: $I_{SR}$ representing infections where sensitive bacteria dominate but resistant bacteria coexist, and, $I_{RS}$ where resistant strains dominate. Transitions into these compartments occur at rates $\eta I_{S}I_{R}$ and $\xi I_{R}I_{S}$, respectively, reflecting asymmetries in colonization success observed in empirical studies [15,23].

**Figure 3:**
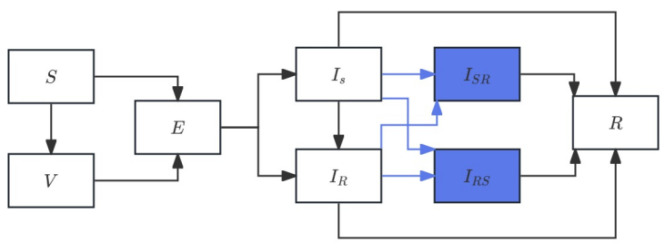
The diagram illustrates the structure of the extended treatment-competition model integrating vaccination, infection progression, treatment-driven resistance, and bidirectional competitive co-colonization. Susceptible individuals (S) may become vaccinated (V) or early carriage establishment (E), progressing to drug-sensitive infection or drug-resistant infection with probability (1−θ) and θ, respectively. Blue arrows indicate competitive interactions between the two strains: co-colonization driven by sensitive-dominant competition produces the state, while resistant-dominant competition produces the state. All infectious states eventually transition to the recovered compartment (R). The schematic highlights the ecological mechanism through which treatment pressure and strain competition jointly shape resistance evolution and coexistence.

A key feature unique to the treatment-competition extension is treatment-induced resistance, represented by the transition $I_{S}$ to $I_{S}$ at rate κτ, capturing the possibility that antibiotic exposure directly generates resistant infections through selection or induction [15,16]. Together, these mechanisms allow the model to reproduce empirically observed patterns such as delayed resistant-strain peaks, coexistence under high treatment pressure, and nonlinear treatment–resistance responses. A schematic representation of the treatment-diversity model is shown in Figure 3.

Compared with the baseline SEVIR model, the treatment-competition extension adds:

1. Treatment-induced conversion from $I_{S}$ to $I_{S}$;
2. Co-colonization compartments $I_{SR}$ and $I_{RS}$;
3. Competitive transition terms $\eta I_{S}I_{R}$ and $\xi I_{R}I_{S}$.

While preserving all other disease progression parameters. The extended system is given by:

(4) $$\left\{\begin{array}{l} \frac{dS}{dt}=-\beta_{S}SI_{S}-\beta_{R}SI_{R}-\nu S\\ \frac{dV}{dt}=\nu S-q(\beta_{S}VI_{S}+\beta_{R}VI_{R})\\ \frac{dE}{dt}=\beta_{S}SI_{S}+\beta_{R}SI_{R}+q(\beta_{S}VI_{S}+\beta_{R}VI_{R})-\sigma E\\ \frac{dI_{S}}{dt}=(1-\theta )\sigma E-\gamma_{s}I_{S}-\tau I_{S}-\eta I_{S}I_{R}\\ \frac{dI_{SR}}{dt}=\eta I_{S}I_{R}-\gamma_{SR}I_{SR}\\ \frac{dI_{RS}}{dt}=\xi I_{R}I_{S}-\gamma_{RS}I_{RS}\\ \frac{dI_{R}}{dt}=\theta \sigma E+\kappa \tau I_{S}-\gamma_{R}I_{R}\\ \frac{dR}{dt}=\gamma_{S}I_{S}+\gamma_{RS}I_{RS}+\gamma_{SR}I_{SR}+\gamma_{R}I_{R} \end{array}\right.$$

This extension introduces explicit frequency-dependent transitions between strain classes.

### 2.3. Empirical Anchoring

This study is intended as a theoretical framework to compare mechanisms by which vaccination and antibiotic use may shape antimicrobial resistance (AMR) dynamics in *Streptococcus pneumoniae*. To strengthen biological plausibility and avoid purely anecdotal scenario choices, we adopted an empirical anchoring strategy when defining scenarios and selecting parameter ranges. Rather than fitting a single country-specific time series, we constrained model settings to remain consistent with well-established empirical patterns reported in pneumococcal surveillance and post-intervention studies.

For the pathogen-diversity (PD) mechanism, pneumococcal populations exhibit marked serotype/lineage heterogeneity and serotype replacement following pneumococcal conjugate vaccine (PCV) introduction, with AMR often concentrated in a subset of serotypes/lineages rather than uniformly distributed. We therefore formulate the PD extension for a general number of circulating serotypes/subtypes D (indexed by j = 1,…,D), and we use D = 3 as a default illustrative setting for visualization; the framework naturally generalizes to arbitrary D. Subtype weights and subtype-specific fitness effects are specified within plausible ranges to reflect heterogeneous lineage contributions and differential AMR potential.

For the treatment-competition (TC) mechanism, empirical and theoretical work suggests that changes in antibiotic intensity may produce nonlinear AMR responses, including situations in which reductions in overall burden are not proportional to reductions in resistant fractions. We capture such effects through within-host competition and selection terms and evaluate outcomes using steady-state prevalence, resistant fraction, and basic reproduction numbers to facilitate transparent comparisons across scenarios. The common parameters and scenario-specific parameters are shown in Table 1A,B.

### 2.4. Mathematical Analysis

**Proposition** **1.** *Let* $R_{0}$* denote the basic reproduction number for the i-th subpopulation in the treatment-diversity model, where the population is divided into D subpopulations based on different antibiotic treatment rates* $\tau_{i}$ *. The basic reproduction number for the i-th subpopulation is given by:*

(5) $$R_{0,i}=(S_{i}^{*}+qV_{i}^{*})[\frac{\beta_{S}(1-\theta )}{\gamma_{S}+\tau_{i}}+\frac{\beta_{R}\theta }{\gamma_{R}}]$$

*The total basic reproduction number for the population is given by:*

(6) $$R_{0}=\underset{i}{\max}R_{0,i}$$

**Proof** **of** **Proposition** **1.** To derive $R_{0}$, we begin by considering the dynamics of the treatment-diversity model for the i-th subpopulation. The model describes the temporal changes in the numbers of susceptible, vaccinated, exposed, and infected individuals. For the calculation of R0,we focus on the infected and exposed compartments, as they contribute to the transmission dynamics. □

Step 1: System Equations and Disease-Free Equilibrium (DFE)

The system of equations governing the dynamics of the model for subpopulation i is as Equation (2). The disease-free equilibrium (DFE) corresponds to the state where no infection is present in any subpopulation. At the DFE, the number of exposed and infected individuals is zero:

(7) $$E_{i}^{*}=I_{S,i}^{*}=I_{R,i}^{*}=R_{i}^{*}=0$$

The equilibrium values of susceptible $S_{i}$ and vaccinated $V_{i}$ are determined by the population dynamics and vaccination parameters, i.e.,

(8) $$S_{i}^{*}+V_{i}^{*}=N_{i}$$

where ${\;N}_{i}$ is the total population of subpopulation i.

Step 2: Next-Generation Matrix (NGM)

Next, we compute the next-generation matrix for the model. The next-generation matrix describes the rate of new infections entering the exposed class $E_{i}$ and the transitions between compartments. The rate of new infections from susceptible and vaccinated individuals is given by:

(9) $$F_{E_{i}}=\beta_{s}(S_{i}^{*}+qV_{i}^{*})I_{S,i}+\beta_{R}(S_{i}^{*}+qV_{i}^{*})I_{R,i}$$

The Jacobian matrix F for the new infections can be written as:

(10) $$F=\left(\begin{array}{cc} \beta_{S}\left(S_{i}^{*}+qV_{i}^{*}\right) & \beta_{R}\left(S_{i}^{*}+qV_{i}^{*}\right)\\ 0 & 0\\ 0 & 0 \end{array}\right)$$

Step 3: Transition Matrix (V)

The transition matrix V describes the rates at which individuals leave the exposed or infected classes. For the i-th subpopulation, these rates are:

(11) $$\nu_{E_{i}}=\sigma E_{i}$$

(12) $$\nu_{I_{S,i}}=(\gamma_{S}+\tau_{i})I_{S,i}-(1-\theta )\sigma E_{i,}$$

(13) $$\nu_{I_{R,i}}=\gamma_{R}I_{R,i}-\theta \sigma E_{i,}$$

The matrix V is then given by:

(14) $$V=\left(\begin{array}{ccc} \sigma & 0 & 0\\ -(1-\theta )\sigma & \gamma_{S}+\tau_{i} & 0\\ {-\theta }_{\sigma } & 0 & \gamma_{R} \end{array}\right)$$

Step 4: Calculation of $R_{0}$

The basic reproduction number $R_{0}$ is obtained by computing the spectral radius of the next-generation matrix. The matrix describes the rate at which infections are transmitted and persist in the population.

After calculating the eigenvalues, the basic reproduction number $R_{0}$ is given by Equation (5). This expression captures the contribution of both sensitive and resistant strains to the overall transmission dynamics in subpopulation i.

The total basic reproduction number $R_{0}$ for the population is the maximum of the reproduction numbers across all subpopulations as Equation (6).

**Proposition** **2.** *The basic reproduction number *$R_{0}$ *of the extended pathogen-diversity model is:*

(15) $$R_{0,i}=(S_{i}^{*}+qV_{i}^{*})\sum_{j=1}^{D}\omega_{j}\beta_{j}[\frac{1-\theta_{j}}{\gamma_{S,j}+\tau }+\frac{\theta_{j}}{\gamma_{R,j}}]$$

**Proposition** **3.** *The basic reproduction number *$R_{0}$ *of the extended treatment-competition model is:*

(16) $$R_{0,i}=(S^{*}+qV^{*})[\frac{\beta_{S}(1-\theta )}{\gamma_{S}+\tau }+\frac{\beta_{R}(\theta +\kappa \frac{\tau }{\gamma R})}{\gamma_{R}}]$$

The proofs of proposition 2 and proposition 3 follow the same reasoning as that of proposition 1 and are therefore omitted here.

### 2.5. Numerical Implementation and Reproducibility

#### 2.5.1. Software and Numerical Integration

All simulations were implemented in Python (NumPy/SciPy) using SciPy’s solve_ivp with adaptive step-size control. Unless otherwise stated, we report solutions on an evenly spaced output grid while allowing the integrator to take internal adaptive steps. Numerical tolerances were set to rtol = 1 × 10^−6^ and atol = 1 × 10^−9^ for all state variables.

To ensure consistent comparison across figures, we used two standardized simulation horizons depending on the purpose of the analysis. Short-horizon runs (used to illustrate transient epidemic responses) were integrated over t ∈ [0, 365] days with 800 output points. Long-horizon runs (used to characterize endemic behavior and long-term intervention responses) were integrated over t ∈ [0, 2000] days with 2000 output points to allow convergence to a quasi-stationary regime. For long-term summaries (e.g., vaccination or treatment scans), “endemic” outcomes were computed as time averages over the final 200 days of the simulation window (i.e., days 1800–2000), including the total infection burden and the resistant fraction.

The default explicit Runge–Kutta method (RK45) was used for non-stiff regimes; for parameter settings exhibiting stiffness or rapid initial transients, a stiff-capable integrator (e.g., LSODA/BDF) was used to improve numerical stability while maintaining the same tolerance settings. In all simulations, we verified numerical plausibility by checking that state variables remained non-negative up to solver tolerance and that total population mass was approximately conserved under demographic turnover. Software versions: Python 3.11; NumPy 1.26; SciPy 1.11; Matplotlib 3.8.

#### 2.5.2. Code Availability

The simulation scripts used to generate the figures are available from the authors upon reasonable request.

## 3. Results

### 3.1. Mechanism Comparison

To evaluate the distinct contributions of the three mechanisms, we identified five stylised epidemiological patterns of pneumococcal resistance that any mechanistic model should be able to reproduce:

P1. Long-term coexistence of sensitive and resistant strains under intermediate antibiotic use;

P2. Reduction in overall infection burden with increasing vaccination, accompanied by a nonlinear response of resistance frequency;

P3. Uneven distribution of resistance across pathogen subtypes, with some lineages carrying disproportionately high burdens;

P4. A paradoxical pattern in which higher treatment intensity decreases total prevalence but increases the proportion of resistant infections;

P5. Persistence of resistance under heterogeneous antibiotic exposure even when the population-average treatment rate is moderate.

These five patterns serve as benchmark criteria to compare the explanatory power of each mechanism.

We next examined each mechanism in isolation to identify which patterns it can reproduce. We summarize the empirical anchors, corresponding model outputs, and supporting evidence for each mechanism in Table 2.

#### 3.1.1. Treatment Diversity Alone

**Figure 4:**
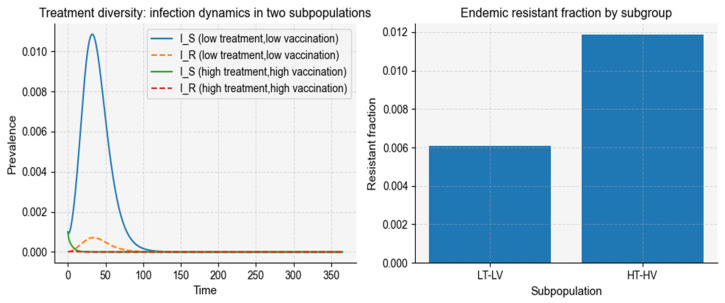
Treatment diversity with correlated vaccination coverage. (Left) Time-series trajectories of drug-sensitive and drug-resistant infections in two subpopulations: a low-treatment/low-vaccination group (LT–LV) and a high-treatment/high-vaccination group (HT–HV). (Right) Endemic resistant fraction in each subgroup, computed as the time-averaged proportion $I_{R}/(I_{S}+I_{R})$ over the final simulation window. The HT–HV subgroup shows a higher resistant fraction than the LT–LV subgroup under the same baseline transmission and natural history parameters, highlighting how correlated heterogeneity in treatment intensity and vaccination coverage can reshape resistance patterns.

When heterogeneity in antibiotic use is introduced across subpopulations, while pathogen natural-history parameters remain homogeneous, the model produces stable coexistence between sensitive and resistant strains under intermediate treatment levels (Figure 4), consistent with empirical observations of resistance persistence in partially treated populations (P1). To better reflect real-world covariation in access to interventions, we allow vaccination coverage to be correlated with treatment intensity across subpopulations (LT–LV vs. HT–HV). Under this correlated heterogeneity, resistance can be maintained even when the population-average treatment intensity is moderate (P5), because the low-treatment/low-vaccination subgroup sustains transmission, whereas the high-treatment/high-vaccination subgroup experiences stronger antibiotic selection, leading to a higher endemic resistant fraction. This LT–LV versus HT–HV representation is empirically motivated by well-documented heterogeneity in healthcare access, where antibiotic exposure and vaccination uptake often covary across subpopulations, thereby sustaining transmission reservoirs while intensifying selection for resistance in highly treated groups. For this parameter set, the population-level basic reproduction number is 1.550.

#### 3.1.2. Pathogen Diversity Alone

**Figure 5:**
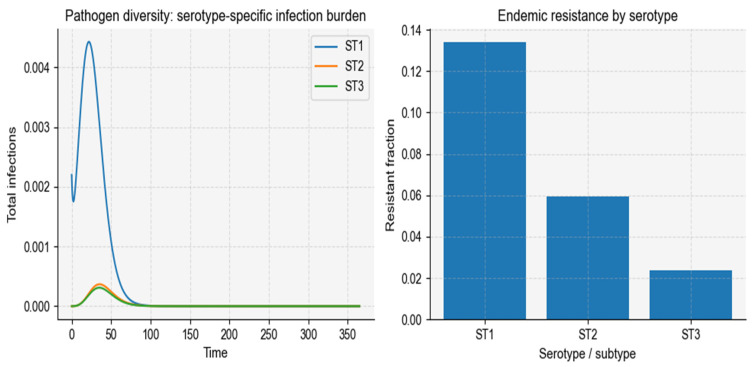
Serotype-specific infection and resistance under pathogen diversity. The figure illustrates how heterogeneity in pathogen serotypes generates uneven infection and resistance burdens. (Left) Time-series trajectories of total infections for three serotypes (ST1–ST3, illustrative labels). The highest-transmission and highest-resistance serotype (ST1) dominates the early epidemic and contributes disproportionately to overall infection burden, while lower-transmission serotypes (ST2 and ST3) produce smaller and shorter outbreaks. (Right) Endemic resistant fractions for each serotype, averaged over the final simulation window. Serotypes with higher resistance emergence probability and slower clearance sustain substantially larger resistant burdens, reproducing the characteristic serotype-specific concentration of resistance observed in pneumococcal populations.

Allowing pathogen subtypes to differ in transmission, resistance propensity, or clearance produces highly uneven distributions of resistance across serotypes/subtypes (Figure 5), closely matching the observed dominance of a few high-resistance clones in pneumococcal populations (P3). We present an illustrative example with D = 3 subtypes to visualize subtype-specific trajectories; the model itself is defined for general. For this PD setting, the computed basic reproduction number is 2.702.

#### 3.1.3. Treatment Competition Alone

**Figure 6:**
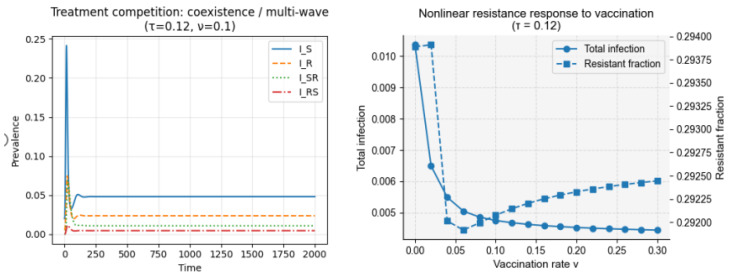
Coexistence dynamics and nonlinear resistance response under treatment competition. The figure demonstrates how treatment-induced competition between sensitive and resistant strains shapes long-term resistance dynamics. (Left) Time-series trajectories of sensitive and resistant infections under intermediate antibiotic treatment and moderate vaccination. Treatment-induced conversion and demographic turnover generate persistent coexistence with characteristic multi-wave dynamics. (Right) Endemic total infection burden and resistant fraction across a range of vaccination rates ν. A nonlinear response emerges: resistance initially increases at low vaccination coverage due to frequency-dependent selection and treatment-induced conversion but decreases once vaccination sufficiently suppresses transmission. Curves represent long-term averages over the final 200 days of simulation.

Introducing within-host competition between sensitive and resistant strains—mediated by treatment-induced conversion (κ) and competitive transitions (η, ξ)—generates nonlinear responses of resistance to antibiotic pressure (Figure 6). Under high treatment, sensitive infections are rapidly cleared, creating ecological space for resistant strains and causing the resistance fraction to increase even as total prevalence declines (P4). This mechanism also produces the delayed rise in resistance following an early sensitive peak, consistent with observed resistance trajectories (P2). Such nonlinear responses—where reductions in overall burden do not translate proportionally into reductions in resistant fractions—have been repeatedly emphasized in evolutionary-epidemiology studies of antibiotic resistance, supporting the plausibility of the treatment-competition mechanism captured here [9,16]. For this set, the computed basic reproduction number is 3.637.

#### 3.1.4. Cross-Mechanism Comparison

To formally assess whether the three mechanisms capture distinct and complementary aspects of resistance evolution, we summarized in Table 3 the ability of each single-mechanism model to reproduce the five key epidemiological patterns (P1–P5). These patterns represent stylised facts commonly observed in pneumococcal resistance: long-term coexistence under intermediate treatment (P1), nonlinear effects of vaccination on resistance (P2), uneven serotype-specific resistance burdens (P3), divergence between total prevalence and resistance fraction under high treatment (P4), and the persistence of resistance under heterogeneous antibiotic exposure (P5).

Taken together, no single mechanism reproduces all five patterns, and each mechanism explains a distinct subset of them. The scenario analysis expands on the implications of Table 3 by showing which mechanisms dominate under different epidemiological conditions.

### 3.2. Scenario Analysis

To examine how different biological mechanisms dominate under distinct epidemiological conditions, we performed a scenario-based comparison using the three extended models described above: the treatment diversity model (TD), the pathogen diversity model (PD), and the treatment competition model (TC). Rather than imposing all mechanisms simultaneously, we evaluated how each model responds to a set of representative intervention scenarios, allowing us to identify which mechanism provides the most plausible explanation in each context. The scenarios were chosen to reflect common real-world settings in pneumococcal epidemiology, focusing on variation in antibiotic treatment intensity, vaccination coverage, population heterogeneity, and pathogen subtype structure.

For each scenario, the three models were simulated under identical baseline parameters except for the focal variables defining the scenario. We compared the predicted trajectories of sensitive and resistant infections, final resistance levels, and qualitative dynamical patterns. The four scenarios are described below.

#### 3.2.1. Scenario A—High Antibiotic Treatment with Minimal Vaccination

Scenario A represents settings with high antibiotic pressure, such as hospital wards or countries with aggressive empirical antibiotic use. In this environment, treatment intensity is sufficiently high to suppress sensitive infections rapidly, thereby altering the competitive balance between sensitive and resistant strains.

**Figure 7:**
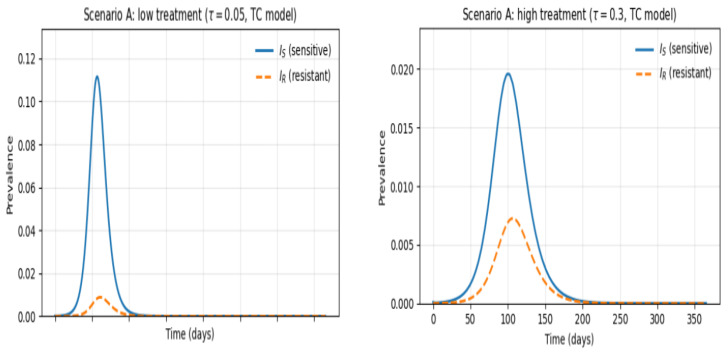
Infection dynamics under low versus high antibiotic treatment. The figure compares infection dynamics under contrasting antibiotic treatment intensities. (Left) Under low treatment, sensitive infections grow rapidly and dominate the epidemic peak, with resistant infections remaining at low levels. (Right) Under high treatment, sensitive infections are strongly suppressed, but resistant infections persist at comparable low levels. Despite different treatment pressures, both scenarios ultimately converge to very low-prevalence equilibria due to the absence of vaccination. Results represent 365-day simulations with identical initial conditions.

Across all simulations, the treatment-competition (TC) mechanism was the only one capable of reproducing the characteristic dynamics observed under heavy antibiotic use. Under TC, early clearance of sensitive infections reduces competition and amplifies the contribution of resistant infections, generating a transient rise in resistance followed by stabilization at a low but persistent level. (Figure 7). This behavior matches empirical observations where aggressive antibiotic treatment reduces overall infections but maintains a measurable resistant component.

In contrast, treatment diversity (TD) and pathogen diversity (PD) produced only attenuated or monotonic responses under strong treatment pressure. TD lacks the inter-strain conversion processes required to sustain resistance when sensitive infections are aggressively removed, while PD exhibits subtype-specific selection but cannot reproduce the rapid early rise in resistant infections characteristic of high-treatment settings.

Overall, Scenario A highlights that high antibiotic pressure accentuates competition between sensitive and resistant strains, making treatment-induced conversion the dominant driver of resistance dynamics. These results underscore the need to reduce unnecessary use of broad-spectrum antibiotics in high-treatment environments, where even short-lived selective advantages can amplify resistant lineages.

#### 3.2.2. Scenario B—Strong Serotype or Lineage Structure

Scenario B examines settings where multiple pathogen serotypes co-circulate, differing in transmissibility and probability of resistance emergence. Such heterogeneity is characteristic of pneumococcal populations, in which only a subset of serotypes contributes disproportionately to overall infection burden and antibiotic resistance.

**Figure 8:**
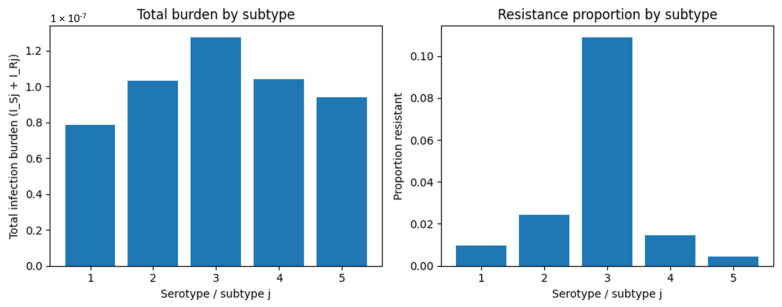
Infection burden and resistant fraction across pathogen subtypes. The figure illustrates how pathogen subtype heterogeneity shapes infection burden and resistance distribution. (Left) Total infection burden across five subtypes, showing substantial differences in transmission potential and outbreak size. (Right) Proportion of resistant infections within each subtype. Subtype 3 exhibits the highest resistance fraction, reflecting its elevated probability of resistance emergence and slower clearance. These results highlight the role of pathogen diversity in concentrating resistance within specific subtypes.

In simulations with three representative serotypes, we observed substantial variation in epidemic magnitude. The highest-transmission serotype produced the earliest and largest peak in total infections, whereas lower-transmission serotypes generated smaller and shorter outbreaks (Figure 8). Resistance was strongly concentrated within the serotype with the highest probability of resistance emergence (θ), which exhibited a resistant fraction more than three-fold greater than that of the other serotypes (Figure 8-right).

To generalize beyond the three-serotype example shown in Figure 8, we extended the analysis to a five-subtype system. This broader evaluation confirmed that resistance consistently concentrates in high-θ subtypes, whereas most subtypes contribute minimally to the resistant pool. The resulting distribution is highly skewed: a small number of high-risk subtypes act as persistent resistance reservoirs, while the majority of subtypes remain predominantly sensitive.

Together, Scenario B demonstrates that pathogen diversity alone can generate strong serotype-specific heterogeneity in resistance. Even modest differences in serotype traits are sufficient to create concentrated resistance burdens in a small subset of high-risk subtypes, mirroring well-documented patterns in pneumococcal serotype ecology.

#### 3.2.3. Scenario C—Heterogeneous Antibiotic Exposure Across Subpopulations

Scenario C represents populations in which antibiotic use is unevenly distributed across subgroups, reflecting common patterns of healthcare access, socioeconomic disparity, or regional prescribing differences. In this setting, we simulated two subpopulations with distinct treatment intensities to evaluate how treatment heterogeneity shapes long-term resistance patterns.

**Figure 9:**
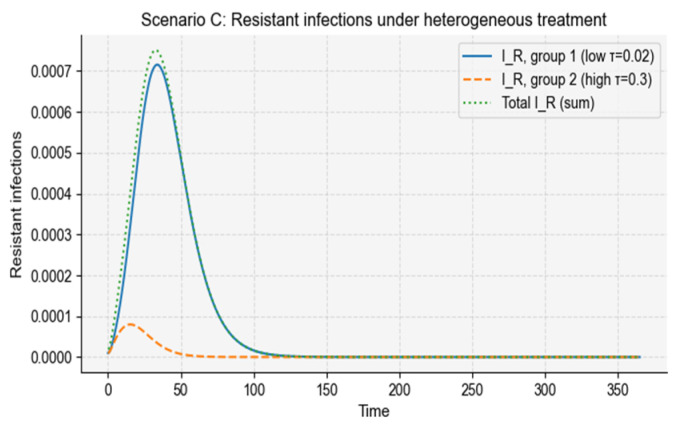
Resistant infection dynamics under heterogeneous antibiotic treatment. The figure shows the temporal evolution of resistant infections in a population composed of two subgroups with distinct treatment rates. Group 1 (low treatment) exhibits a larger peak of resistant infections due to slower clearance and reduced selection pressure on sensitive strains. Group 2 (high treatment) shows a smaller but earlier peak of resistant infections because treatment rapidly suppresses sensitive infections and increases the contribution of resistant infections to total prevalence. The aggregate resistant infections (dotted curve) reflect the combined contribution of both groups, illustrating how heterogeneous treatment environments sustain resistance even when overall treatment is moderate.

Across simulations, the low-treatment subgroup consistently sustained a larger burden of resistant infections, producing both a higher peak and a broader epidemic curve (Figure 9). Reduced antibiotic pressure in this subgroup slows the clearance of sensitive infections, allowing prolonged transmission and increased opportunities for resistance to persist. Conversely, the high-treatment subgroup exhibited a smaller but earlier resistant peak, reflecting rapid suppression of sensitive infections and short-lived selective amplification of resistant ones.

**Figure 10:**
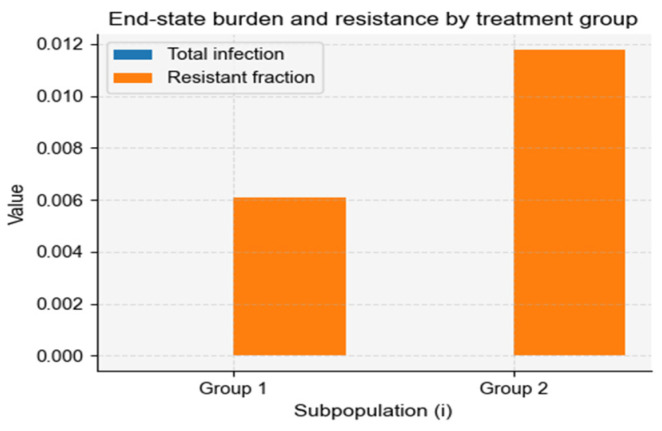
End-state infection burden and resistance across heterogeneous treatment groups. The figure summarizes the final infection burden and resistance proportion in two subpopulations experiencing different antibiotic treatment intensities. Group 1 receives low treatment, resulting in a smaller total infection burden but a moderate resistant fraction. Group 2 receives high treatment, producing a larger resistant proportion despite similar overall infection levels. These results highlight how heterogeneous treatment rates create asymmetric selection pressures that sustain resistance in low-treatment “reservoir” groups while amplifying resistance in high-treatment groups.

When aggregated, the overall resistant burden remained elevated despite moderate average treatment, driven predominantly by the contribution of the low-treatment subgroup (Figure 10). This asymmetric contribution demonstrates how heterogeneous treatment creates “resistance reservoirs” that can maintain resistance at the population level even when high-treatment groups are individually well controlled.

Thus, Scenario C shows that uneven antibiotic use amplifies population-level resistance by sustaining transmission in inadequately treated subgroups, making treatment heterogeneity a key driver of persistent resistance in real-world settings.

#### 3.2.4. Scenario D—Intermediate Treatment and Moderate Vaccination

Scenario D explores a setting with sustained demographic turnover and simultaneous vaccination and antibiotic treatment, representing long-term community-level control efforts. This scenario evaluates how vaccination coverage interacts with treatment pressure to shape resistance dynamics.

**Figure 11:**
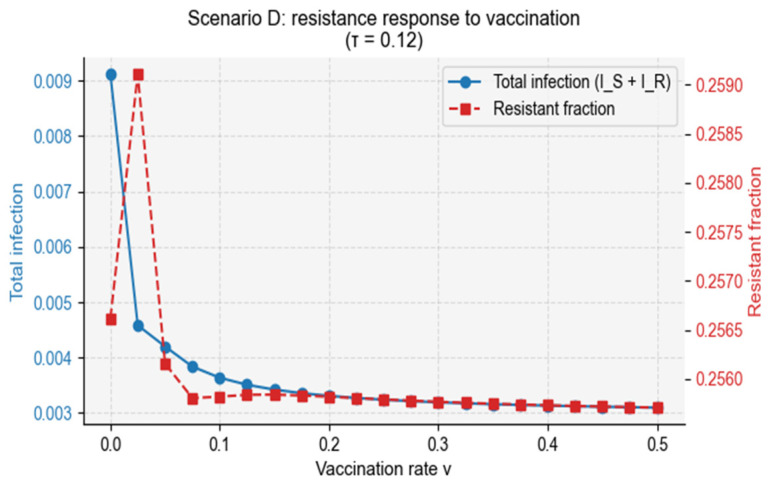
Nonlinear resistance response to vaccination under treatment competition. The figure displays the endemic infection burden and resistant fraction across a range of vaccination rates under treatment competition. (Left axis) Total infection burden declines monotonically with increasing vaccination coverage due to reduced transmission. (Right axis) The resistant fraction exhibits a nonlinear pattern: resistance initially increases at low vaccination coverage, driven by frequency-dependent selection and treatment-induced conversion, and subsequently declines once vaccination sufficiently suppresses overall infection levels. Values represent long-term averages computed over the final 200 days of simulation.

Under intermediate treatment and moderate vaccination (τ = 0.12, ν = 0.10), simulations produced stable coexistence of sensitive and resistant infections at low prevalence (Figure 11). Birth and death processes continuously replenish susceptible individuals, allowing repeated waves of sensitive infections, while treatment-induced conversion maintains a persistent resistant component. This interaction generates multi-wave dynamics in which resistance never fully disappears but remains at low equilibrium levels.

**Figure 12:**
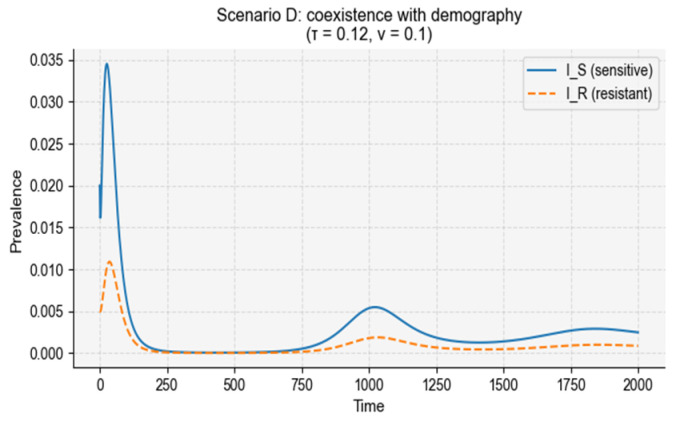
Long-term coexistence of sensitive and resistant strains under demographic turnover. The figure shows the temporal dynamics of sensitive and resistant infections under intermediate treatment, moderate vaccination, and demographic turnover. Continuous birth and death processes replenish the susceptible pool, enabling long-term coexistence between sensitive and resistant strains. Multiple secondary waves emerge due to the interaction between demographic renewal and treatment-induced conversion, with resistant infections consistently present at low but persistent levels throughout the simulation period of 2000 days.

Scanning across vaccination rates revealed a nonlinear resistance response (Figure 12). Total infection burden declined monotonically as vaccination reduced transmission, but the resistant fraction followed a non-monotonic trajectory: resistance initially increased at low vaccination coverage, driven by frequency-dependent selection and treatment-induced conversion, before decreasing once vaccination sufficiently suppressed overall transmission. This pattern mirrors empirical observations where partial vaccination can inadvertently increase resistance, while high coverage eventually suppresses it.

Together, these results show that the interplay between treatment pressure, vaccination, and demographic renewal produces complex resistance dynamics, highlighting the importance of jointly optimizing antibiotic use and vaccine coverage to minimize resistance.

## 4. Discussion

The unified SEVIR-based framework developed in this study highlights that the interaction between vaccination and antimicrobial resistance (AMR) cannot be attributed to a single ecological or behavioral mechanism. By embedding three widely discussed processes—treatment diversity, pathogen diversity, and treatment competition—within a common dynamical structure and comparing them under controlled scenarios, we show that qualitatively different resistance trajectories can emerge from distinct mechanisms. In particular, heterogeneous antibiotic exposure sustains resistance under moderate average use; subtype/serotype heterogeneity concentrates resistance within specific lineages; and within-host competition under treatment produces nonlinear and sometimes counterintuitive population-level responses. Taken together, these results provide a coherent mechanistic map linking intervention levers (vaccination and antibiotic use) to several empirically observed patterns of pneumococcal resistance dynamics.

### 4.1. Mechanistic Interpretation and Empirical Anchors

First, the treatment-diversity (TD) mechanism illustrates how unequal access to antibiotics across subpopulations can maintain resistant strains even when population-average use is not high: low-treatment groups can function as reservoirs supporting prolonged circulation, while higher-treatment groups impose stronger selective pressure that increases the relative resistant fraction. This pattern is consistent with observations that resistance persistence is often driven by heterogeneity in antibiotic exposure rather than mean consumption alone. Our findings have several implications for public health practice. First, resistance persistence under moderate overall antibiotic use is primarily driven by treatment heterogeneity, indicating that low-treatment subpopulations serve as long-term reservoirs sustaining both sensitive and resistant strains [24]. Second, resistance concentration in specific serotypes reflects intrinsic pathogen heterogeneity, suggesting that vaccine formulations must anticipate serotype replacement and shifting resistance burdens [25]. Third, counterintuitive patterns—such as decreased total infections but increased resistance under intensified treatment—emerge naturally from treatment competition, emphasizing that increasing antibiotic pressure alone may exacerbate resistance even while reducing disease burden. These insights explain why population-level resistance trends often respond nonlinearly or unpredictably to large-scale interventions.

Second, the pathogen-diversity (PD) mechanism captures the fact that pneumococcal populations are structured into multiple serotypes/subtypes that differ in transmissibility, duration of carriage, and resistance propensity. Under vaccination that targets only part of the circulating diversity, the framework reproduces serotype replacement and shifting resistance burdens toward non-vaccine types, as reported in multiple post-PCV settings. This provides a plausible explanation for why resistance may decrease in vaccine-covered serotypes while concentrating or rising in specific non-vaccine lineages in the longer term.

Third, the treatment-competition (TC) mechanism emphasizes that within-host competition and treatment-induced selection can yield nonlinear relationships between antibiotic pressure, overall prevalence, and resistance fraction. In these scenarios, intensified treatment may reduce total infections but transiently increase the resistant proportion, illustrating that “lower burden” does not necessarily translate into “lower resistance”. Such nonlinearities have been repeatedly highlighted in evolutionary-epidemiology studies of AMR and can help interpret apparently paradoxical surveillance trends.

### 4.2. Public Health Implications

These findings suggest that effective resistance control requires coordinated strategies that target multiple mechanisms simultaneously. Reducing treatment heterogeneity—through improved access, rational prescribing, and ensuring appropriate therapy in under-treated groups—may disrupt reservoir effects that sustain transmission. Vaccine design and deployment should explicitly anticipate serotype replacement and potential shifts in resistance burden across lineages, motivating continuous surveillance and flexible vaccine composition updates where feasible. Finally, antibiotic stewardship should be evaluated not only for its effect on total disease incidence but also for its effect on selection and competitive dynamics that can amplify resistant fractions under certain regimes. Overall, integrated policies combining vaccination strategy, antibiotic-use optimization, and targeted interventions for high-risk populations are more likely to achieve durable reductions in AMR than single-lever approaches.

### 4.3. Model Scope and Limitations

Two modeling choices deserve clarification in relation to pneumococcal biology. First, pneumococcal colonization can become transmissible soon after acquisition; thus, a strict “latent, non-infectious” period is not always appropriate. In our framework, the exposed class E should be interpreted as a short establishment/detection stage between acquisition and established carriage, included primarily for structural convenience and comparability across scenarios rather than as a claim of a long biological latency. Future work could replace this component with an SIS-type carriage structure, or introduce partially infectious early carriage stages, when detailed acquisition-to-carriage data are available.

Second, pneumococcal transmission is largely driven by asymptomatic carriage, while antibiotic exposure is concentrated among symptomatic disease episodes. Our current structure does not explicitly separate carriage from disease; accordingly, the treatment term τ should be interpreted as an effective, population-level antibiotic selection pressure acting on the circulating pneumococcal population, rather than literal treatment applied to all colonized individuals. Extending the model to a carriage–disease structure—where treatment acts only on symptomatic compartments while carriage drives most transmission—represents an important direction for improving biological realism and strengthening links to clinical antibiotic-use data.

## 5. Conclusions

In this study, we developed a unified dynamical framework to investigate how vaccination, antibiotic treatment, and strain resistance interact to shape pneumococcal transmission. Building on a baseline SEVIR model, we incorporated three mechanistic extensions—treatment diversity, pathogen diversity, and treatment competition—that represent distinct ecological and behavioral drivers of resistance. Through analytical derivations, we established the basic reproduction numbers for each extended system and characterized conditions for disease-free equilibrium and the coexistence of sensitive and resistant strains.

Using this framework, we systematically evaluated the capacity of each mechanism to reproduce five empirically observed resistance patterns. Our results demonstrate that no single mechanism can capture all patterns; instead, each mechanism generates a unique subset of observed behaviors. Treatment diversity explains the persistence of resistance under heterogeneous antibiotic use and supports long-term coexistence of strains. Pathogen diversity accounts for the highly uneven distribution of resistance across serotypes. Treatment competition gives rise to nonlinear responses to vaccination and antibiotic pressure, as well as scenarios where overall infections decline while resistance increases.

Finally, scenario-based simulations show that the dominant mechanism driving resistance can shift across epidemiological and intervention contexts. Collectively, these findings highlight the utility of a unified mechanistic model for disentangling the multiple forces that shape resistance dynamics and for identifying the specific conditions under which each mechanism becomes most influential.

## Figures and Tables

**Table 1:** (**A**) Common parameters across all scenarios. (**B**) Scenario-specific parameters.

**(A) Common Parameters Across All Scenarios**			
**Symbol**	**Definition**	**Value**	**Note**
σ	progression rate from the exposed to the infectious class	0.250	Fixed; adopted from published SEIR/SEVIR modeling literature [2].
θ	probability of resistance emergence	0.060	Fixed; assumed resistance emergence probability per treatment event.
$\gamma_{S}$	natural recovery rate for drug-sensitive infections	0.056	Based on pneumococcal carriage duration/clearance assumptions in dynamic transmission models [8].
$\gamma_{R}$	natural recovery rate for drug-resistant infections	0.042	Based on pneumococcal carriage duration/clearance assumptions in dynamic transmission models [8].
q	vaccine failure rate	0.500	Fixed; vaccine failure/breakthrough factor; assumed (q = 0.5).
ν	vaccination coverage	Scenario-dependent	Varied by scenario; values reported in Table 1B
$\beta_{S}$	transmission rate of the drug-sensitive strain	calibrated	$\mathrm{Calibrated}\;\mathrm{to}\;R_{0}$
$\beta_{R}$	transmission rate of the drug-resistant strain	calibrated	$\mathrm{Set}\;\mathrm{relative}\;\mathrm{to}\;\beta_{S}$$[\mathrm{assumed}\;\mathrm{ratio}]\;(\beta_{R}$$=\;k\text{·}\beta_{R}$)
τ	effective treatment pressure	Scenario-dependent	Varied by scenario; baseline and alternative treatment levels are listed in Table 1B
**(B) Scenario-Specific Parameters**			
**Symbol**	**Definition**	**Value**	**Note**
$\tau_{i}$	antibiotic treatment rate in subgroup i	{0.05, 0.20}	TD
$\nu_{i}$	vaccination coverage in subgroup i	{0.20, 0.80}	TD
$\theta_{i}$	probability of resistance emergence in subgroup i	{0.03, 0.09}	TD
$\omega_{j}$	population weight of subtype j	{0.33, 0.33, 0.34}	PD
$\beta_{j}$	transmission rate of subtype j	{0.26, 0.30, 0.34}	PD
$c_{j}$	fitness cost of resistant subtype j	{0.00, 0.10, 0.20}	PD
$\theta_{j}$	probability of resistance emergence for subtype j	0.060	PD
$\gamma_{S,j}$	natural recovery rate for drug-sensitive infections of subtype j	0.056	PD
$\gamma_{R,j}$	natural recovery rate for drug-resistant infections of subtype j	0.100	PD
κ	treatment-induced conversion rate	0.25	TC
η	conversion from sensitive to resistant	0.30	TC
ξ	reverse conversion	0.10	TC
$\gamma_{SR}$	$\mathrm{recovery}\;\mathrm{rate}\;\mathrm{for}\;\mathrm{class}\;I_{SR}$	0.056	TC
$\gamma_{RS}$	$\mathrm{recovery}\;\mathrm{rate}\;\mathrm{for}\;\mathrm{class}\;I_{RS}$	0.100	TC

**Table 2:** Empirical anchoring of the three mechanistic extensions.

Mechanism	Empirical Anchor	Model Output Used for Comparison	Evidence
Treatment Diversity	Resistance can persist under moderate population-average antibiotic use when treatment is heterogeneous across subpopulations	Endemic resistant fraction by subgroup; persistence of resistance when τand ν are moderate; subgroup-specific infection burden and resistance.	Global AMR burden context and treatment heterogeneity/access disparities discussion [1]
Pathogen Diversity	Post-PCV era shows serotype replacement and shifts in resistance burden concentrated in particular serotypes/lineages, reflecting serotype-specific fitness and resistance propensity.	Serotype-specific infection burden and resistant fraction; replacement dynamics; distribution (skew) of resistance across serotypes.	Serotype replacement after pneumococcal conjugate vaccination [4]; post-PCV serotype/lineage dynamics and resistance redistribution ref; frequency-dependent selection/serotype fitness heterogeneity [23].
Treatment Competition	Antibiotic intensification can yield nonlinear resistance responses—overall burden decreases but resistant fraction may transiently increase or rebound due to within-host selection/competition.	Resistance fraction vs. treatment intensity curves; transient resistance surges; delayed resistant rise after an early sensitive peak.	Within-host dynamics shaping resistance under treatment and competition frameworks [15]; evolutionary ecology/treatment-competition modeling evidence [17]

Note. Anchors are used to constrain parameter ranges and to interpret qualitative patterns; the framework is not intended as a country-specific forecasting model.

**Table 3:** Ability of three mechanisms to reproduce empirical resistance patterns.

Mechanism	P1	P2	P3	P4	P5
Treatment Diversity	√	×	×	-	√
Pathogen Diversity	-	×	√	×	×
Treatment Competition	√	-	×	√	×

√ = strong reproduction of empirical pattern; - = partial/weak reproduction; × = mechanism cannot generate this pattern.

## Data Availability

The original contributions presented in this study are included in the article. Further inquiries can be directed to the corresponding author.
